# Supplementary material for: Homogeneous surrogate virus neutralization assay to rapidly assess neutralization activity of anti-SARS-CoV-2 antibodies
Source: Nat Commun. 2022 Jul 1;13:3716. doi: 10.1038/s41467-022-31300-9 (PMC9249905; doi:10.1038/s41467-022-31300-9)
Supplement: Supplementary file 1 — Supplementary Information [file 41467_2022_31300_MOESM1_ESM.pdf]

Supplementary information to accompany

Homogeneous Surrogate Virus Neutralization Assay to Rapidly Assess Neutralization Activity of Anti-SARS-CoV-2 Antibodies

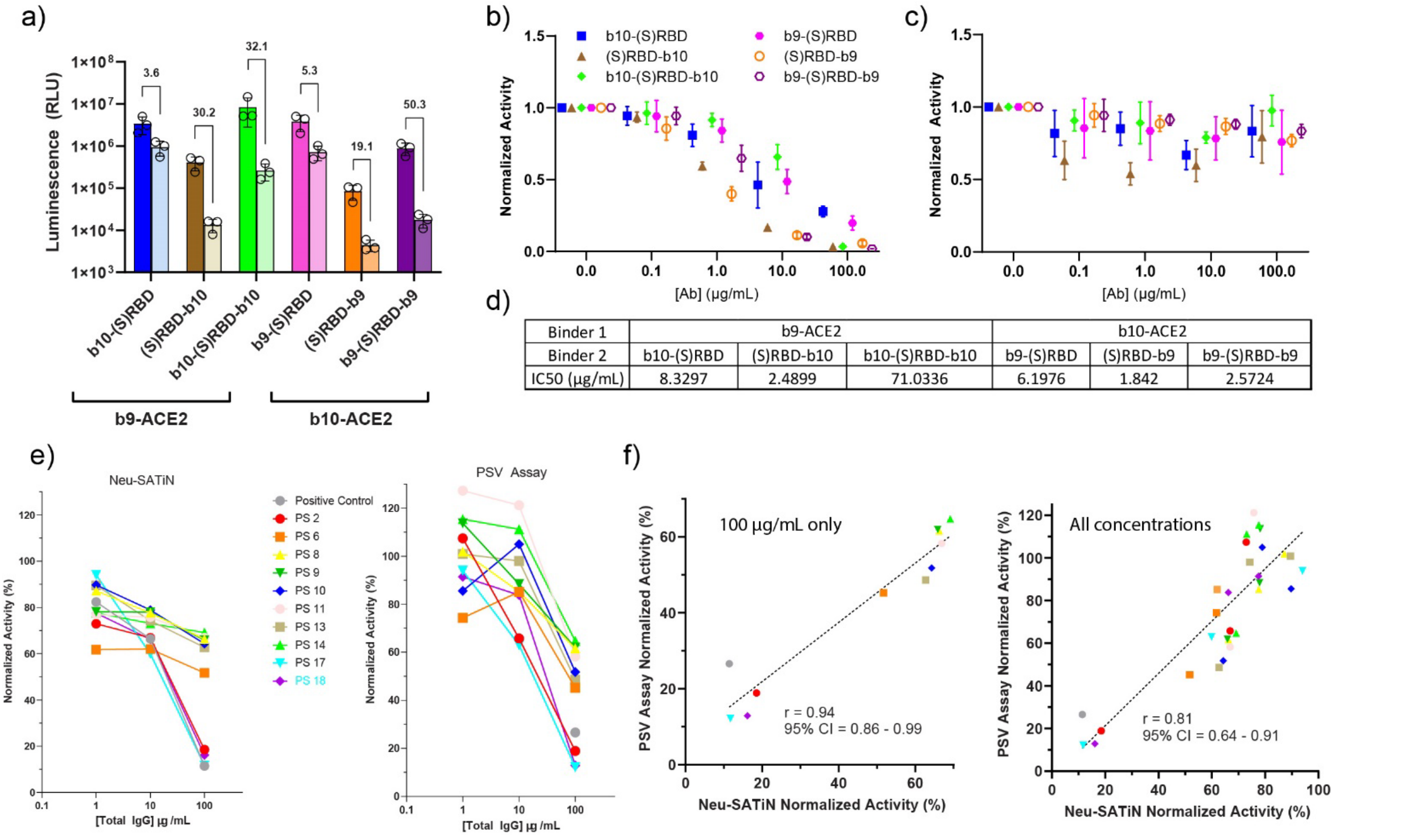

d)

Binder 1	b9-ACE2			b10-ACE2		
Binder 2	b10-(S)RBD	(S)RBD-b10	b10-(S)RBD-b10	b9-(S)RBD	(S)RBD-b9	b9-(S)RBD-b9
IC50 (μg/mL)	8.3297	2.4899	71.0336	6.1976	1.842	2.5724

Supplementary Figure 1. Specificity of RBD binder pair in simulated human serum and IC50 values of each binder pair. a) Comparison of signals between 0 μg/mL of NAb (darker color) vs. 100 μg/mL of NAb (lighter color adjacent bar) from six different pairs. Although all pairs show good response, some pairs show more distinct fold-difference between without vs. with NAb as indicated by numbers above bars. **b)** Pairs of ACE2 binders and (S)RBD binders were screened with increasing concentrations of neutralizing Ab (NAb, Sino Biological 40592-MM57) spiked in human serum. All pairs show substantial decrease in signal with increasing concentrations of NAb, demonstrating a quantifiable dose-response. **c)** When each binder pair was incubated with control isotype IgG-spiked human serum (additional concentrations of IgG spiked into human serum), less than 20% inhibition from high IgG (100 μg/mL) was observed in all of the pairs. **d)** IC50 (μg/mL) of the same NAb was calculated using six different binder pairs with AAT Bioquest IC50 calculator. **e)** Representative samples from Fig. 2d were serially diluted and tested by Neu-SATiN (left). As this in-house PSV assay is sensitive to protein content, performing the assay using direct plasma samples was not feasible. Instead, we selected ten samples (non-black bars on Fig. 2d) and extracted plasma IgG using protein G magnetic beads. The purified samples were serially diluted and tested with PSV assay (right). Each assay was repeated four individual times (n = 4, biologically independent repeats) and the samples were run in triplicate (technical repeats). Average activity is shown (symbols). **f)** Average measurements were plotted and highest concentrations (left) show high correlation between the two assays with an r value of 0.94 and good correlation across all dilutions (right) with an r value of 0.81. NAb was used as an internal positive control (gray). One potential source of discrepancy seen between the results is lack of IgMs in PSV assay. Protein G is effective only in purifying IgGs, therefore purified clinical plasma mainly contained IgGs to be tested on PSV assay. As Neu-SATiN is performed directly using whole plasma, the antiviral activities of both IgGs and IgMs may contribute to the level of inhibition.[50] Data (a-c) are presented as mean values with error bars indicating SD. Source data are provided as a Source Data file.

Supplementary Table 1. Amino acid sequences of the binders. Spike protein and ACE2 sequences were obtained from literature sources. Then, each binder was PCR-modified with appropriate tags and linkers to produce binders. Octa-histidine was used as the purification tag. M denotes the location of the START codon and * denotes STOP codon. PDGFRB signal peptide (RLPGAMPALALKGELLLLSLLLLLEPQISQG) was used to promote protein secretion. The sequences for b9 and b10 are GSMLFRVTINS and VSGWRFKKIS, respectively.

Binder	Tag Orientation	Sequence	Note
(S)RBD	b9-(S)RBD	MRLPGAMPALALKGELLLLSLLLLLEPQISQGGGGSMLFRVTINSGGGGSGGGSRVQPTESIVRFPNITNLCPFGEVFNATRFASVYAWNRKRISNCVADYSVLYNSASFSTFKCYGVSPTKLNDLCFTNVYADSFVIRGDEVRQIAPGQTGKIADYNYKLPDDFTGCVIAWNSNNLDSKVGGNYNYLYRLFRKSNLKPFERDISTEIYQAGSTPCNGVEGFNCYFPLQSYGFQPTNGVGYQPYRVVVLSFELLHAPATVCGPKKSTNLVKNKCVNFGGSHHHHHHHH*	
	(S)RBD-b9	MFVFLVLLPLVSSQRVQPTESIVRFPNITNLCPFGEVFNATRFASVYAWNRKRISNCVADYSVLYNSASFSTFKCYGVSPTKLNDLCFTNVYADSFVIRGDEVRQIAPGQTGKIADYNYKLPDDFTGCVIAWNSNNLDSKVGGNYNYLYRLFRKSNLKPFERDISTEIYQAGSTPCNGVEGFNCYFPLQSYGFQPTNGVGYQPYRVVVLSFELLHAPATVCGPKKSTNLVKNKCVNFGGGGSGGGSGSMLFRVTINSGGSHHHHHHHH*	
	b9-(S)RBD-b9	MRLPGAMPALALKGELLLLSLLLLLEPQISQGGGGSMLFRVTINSGGGGSGGGSRVQPTESIVRFPNITNLCPFGEVFNATRFASVYAWNRKRISNCVADYSVLYNSASFSTFKCYGVSPTKLNDLCFTNVYADSFVIRGDEVRQIAPGQTGKIADYNYKLPDDFTGCVIAWNSNNLDSKVGGNYNYLYRLFRKSNLKPFERDISTEIYQAGSTPCNGVEGFNCYFPLQSYGFQPTNGVGYQPYRVVVLSFELLHAPATVCGPKKSTNLVKNKCVNFGGGGSGGGSGSMLFRVTINSGGSHHHHHHHH*	
	b10-(S)RBD	MRLPGAMPALALKGELLLLSLLLLLEPQISQGGGVSGWRLFKKISGGGGSGGGSRVQPTESIVRFPNITNLCPFGEVFNATRFASVYAWNRKRISNCVADYSVLYNSASFSTFKCYGVSPTKLNDLCFTNVYADSFVIRGDEVRQIAPGQTGKIADYNYKLPDDFTGCVIAWNSNNLDSKVGGNYNYLYRLFRKSNLKPFERDISTEIYQAGSTPCNGVEGFNCYFPLQSYGFQPTNGVGYQPYRVVVLSFELLHAPATVCGPKKSTNLVKNKCVNFGGSHHHHHHHH*	
	(S)RBD-b10	MFVFLVLLPLVSSQRVQPTESIVRFPNITNLCPFGEVFNATRFASVYAWNRKRISNCVADYSVLYNSASFSTFKCYGVSPTKLNDLCFTNVYADSFVIRGDEVRQIAPGQTGKIADYNYKLPDDFTGCVIAWNSNNLDSKVGGNYNYLYRLFRKSNLKPFERDISTEIYQAGSTPCNGVEGFNCYFPLQSYGFQPTNGVGYQPYRVVVLSFELLHAPATVCGPKKSTNLVKNKCVNFGGGGSGGGSVSGWRLFKKISGGSHHHHHHHH*	
	b10-(S)RBD-b10	MRLPGAMPALALKGELLLLSLLLLLEPQISQGGGVSGWRLFKKISGGGGSGGGSRVQPTESIVRFPNITNLCPFGEVFNATRFASVYAWNRKRISNCVADYSVLYNSASFSTFKCYGVSPTKLNDLCFTNVYADSFVIRGDEVRQIAPGQTGKIADYNYKLPDDFTGCVIAWNSNNLDSKVGGNYNYLYRLFRKSNLKPFERDISTEIYQAGSTPCNGVEGFNCYFPLQSYGFQPTNGVGYQPYRVVVLSFELLHAPATVCGPKKSTNLVKNKCVNFGGGGSGGGSVSGWRLFKKISGGSHHHHHHHH*	
Spike (Full)	b10-Spike (18 G/S linker)	MRLPGAMPALALKGELLLLSLLLLLEPQISQGGGVSGWRLFKKISGGGGSGGGSGGGGSGGGSCVNLTTRTQLPPAYTNSFTRGVYYPDKVFRSSVLHSTQDLFLPFFSNVTWFHAIHVSGTNGTKRFDNPVLPFNDGVYFASTEKSNIIRGWIFGTTLDSKTQSLLIVNNATNVVIKVCEFQFCNDPFLGVYYHKNNKSWMESEFRVYSSANNCTFEYVSQPFLMDLEGKQGNFKNLREFVFKNIDGYFKIYSKHTPINLVRDLPQGFSALEPLVDLPIGINITRFQTLLALHRSYLTPGDSSSGWTAGAAAYYVGYLQPRTFLLKYNENGTITDAVDCALDPLSETKCTLKSFTVEKGIYQTSNFRVQPTESIVRFPNITNLCPFGEVFNATRFASVYAWNRKRISNCVADYSVLYNSASFSTFKCYGVSPTKLNDLCFTNVYADSFVIRGDEVRQIAPGQTGKIADYNYKLPDDFTGCVIAWNSNNLDSKVGGNYNYLYRLFRKSNLKPFERDISTEIYQAGSTPCNGVEGFNCYFPLQSYGFQPTNGVGYQPYRVVVLSFELLHAPATVCGPKKSTNLVKNKCVNFNFNGLTGTGVLTESNKKFLPFQQFGRDIADTTDAVRDPQTLEILDITPCSFGGVSVITPGTNTSNQVAVLYQDVNCTEVPVAIHADQLTPTWRVYSTGSNVFQTRAGCLIGAEHVNNSYECDIPIGAGICASYQTQTNSPGSASSVASQSIIAYTMSLGAENSVAYSNNSIAIPTNFTISVTTEILPVSMTKTSVDCTMYICGDSTECSNLLLQYGSFCTQLNRALTGIAVEQDKNTQEVFAQVKQIYKTPPIKDFGGFNFSQILPDPSKPSKRSFIEDLLFNKVTLADAGFIKQYGDCLGDIAARDLICAQKFNGLTVLPPLLTDEMIAQYTSALLAGTITSGWTFGAGAALQIPFAMQMAYRFNGIGVTQNVLYENQKLIANQFNSAIGKIQDSLSSTASALGKLQDVVNQNAQALNTLVKQLSSNFGAISSVLNDILSRLDPPEAEVQIDRLITGRLQSLQTYVTQQLIRAAEIRASANLAATKMSECVLGQSKRVDFCGKGYHLMSFPQSAPHGVVFLHVTYVPAQEKNFTTAPAICHDGKAHFPREGVFVSNGTHWFVTQRNFYEPQIITTDNTFVSGNCDVVIGIVNNTVYDPLQPELDSFKEELDKYFKNHTSPDVDLGDISGINASVVNIQKEIDRLNEVAKNLNESLIDLQELGKYEQYIKWPGGSGGYIPEAPRDGQAYVRKDGEWVLLSTFLGGSHHHHHHHH*	
ACE2	b9-ACE2 (9 G/S linker)	MRLPGAMPALALKGELLLLSLLLLLEPQISQGGGGSMLFRVTINSGGGGSGGGSAAQSTIEEQAKTFLDKFNHEAEDLFYQSSLASWNYNTNITEENVQNMNNAGDKWSAFLKEQSTLAQMYPLQEIQNLTVKLQLQALQQNGSSVLSEDKSKRLNTILNTMSTIYSTGKVCNPDNPQECLLLEPGLNEIMANSLDYNERLWAWESWRSEVGKQLRPLYEEYVVLKNEMARANHYEDYGDYWRGDYEVNGVDGYDYSRGQLIEDVEHTFEEIKPLYEHLHAYVRAKLMNAYPSYISPIGCLPAHLLGDMWGRFWTNLYSLTVPFGQKPNIDVTDAMVDQAWDAQRIFKEAEKFFVSVGLPNMTQGFWENSMLTDPGNVQKAVCHPTAWDLGKGDFRILMCTKVTMDDFLTAHHEMGHIQYDMAYAAQPFLLRNGANEGFHEAVGEIMSLSAATPKHLKSIGLLSPDFQEDNETEINFLLKQALTIVGTLPFTYMLEKWRWMVFKGEIPKDQWMKKWWEMKREIVGVVEPVPHDETYCDPASLFHVSNDYSFIRYYTRTLYQFQFQEALCQAAKHEGPLHKCDISNSTEAGQKLFNMLRLGKSEPWTLALENVVGAKNMNVRPLLNYFEPLFTWLKDQNKNSFVGWSTDWSPYAGGSHHHHHHHH*	used in pair with (S)RBD
	b10-ACE2 (9 G/S linker)	MRLPGAMPALALKGELLLLSLLLLLEPQISQGGGVSGWRLFKKISGGGGSGGGSAAQSTIEEQAKTFLDKFNHEAEDLFYQSSLASWNYNTNITEENVQNMNNAGDKWSAFLKEQSTLAQMYPLQEIQNLTVKLQLQALQQNGSSVLSEDKSKRLNTILNTMSTIYSTGKVCNPDNPQECLLLEPGLNEIMANSLDYNERLWAWESWRSEVGKQLRPLYEEYVVLKNEMARANHYEDYGDYWRGDYEVNGVDGYDYSRGQLIEDVEHTFEEIKPLYEHLHAYVRAKLMNAYPSYISPIGCLPAHLLGDMWGRFWTNLYSLTVPFGQKPNIDVTDAMVDQAWDAQRIFKEAEKFFVSVGLPNMTQGFWENSMLTDPGNVQKAVCHPTAWDLGKGDFRILMCTKVTMDDFLTAHHEMGHIQYDMAYAAQPFLLRNGANEGFHEAVGEIMSLSAATPKHLKSIGLLSPDFQEDNETEINFLLKQALTIVGTLPFTYMLEKWRWMVFKGEIPKDQWMKKWWEMKREIVGVVEPVPHDETYCDPASLFHVSNDYSFIRYYTRTLYQFQFQEALCQAAKHEGPLHKCDISNSTEAGQKLFNMLRLGKSEPWTLALENVVGAKNMNVRPLLNYFEPLFTWLKDQNKNSFVGWSTDWSPYAGGSHHHHHHHH*	used in pair with (S)RBD
	b9-ACE2-Fc (18 G/S linker)	MRLPGAMPALALKGELLLLSLLLLLEPQISQGGGGSMLFRVTINSGGGGSGGGSGGGGSGGGSAAQSTIEEQAKTFLDKFNHEAEDLFYQSSLASWNYNTNITEENVQNMNNAGDKWSAFLKEQSTLAQMYPLQEIQNLTVKLQLQALQQNGSSVLSEDKSKRLNTILNTMSTIYSTGKVCNPDNPQECLLLEPGLNEIMANSLDYNERLWAWESWRSEVGKQLRPLYEEYVVLKNEMARANHYEDYGDYWRGDYEVNGVDGYDYSRGQLIEDVEHTFEEIKPLYEHLHAYVRAKLMNAYPSYISPIGCLPAHLLGDMWGRFWTNLYSLTVPFGQKPNIDVTDAMVDQAWDAQRIFKEAEKFFVSVGLPNMTQGFWENSMLTDPGNVQKAVCHPTAWDLGKGDFRILMCTKVTMDDFLTAHHEMGHIQYDMAYAAQPFLLRNGANEGFHEAVGEIMSLSAATPKHLKSIGLLSPDFQEDNETEINFLLKQALTIVGTLPFTYMLEKWRWMVFKGEIPKDQWMKKWWEMKREIVGVVEPVPHDETYCDPASLFHVSNDYSFIRYYTRTLYQFQFQEALCQAAKHEGPLHKCDISNSTEAGQKLFNMLRLGKSEPWTLALENVVGAKNMNVRPLLNYFEPLFTWLKDQNKNSFVGWSTDWSPYAGGSGGGGSGGGGSTCPPCPAPELLGGPSVFLFPPKPKDTLMISRTPEVTCVVVDVSHEDPEVKFNWYVDGVEVHNAKTKPREEQYNSTYRVVSVLTVLHQDWLNGKEYKCKVSNKALPAPIEKTISKAKGQPREPQVYTLPPSRDELTKNQVSLTCLVKGFYPSDIAVEWESNGQPENNYKTTPPVLDSDGSFFLYSKLTVDKSRWQQGNVFSCSVMHEALHNHYTQKSLSLSPGKGGSHHHHHHHH*	used in pair with (S)WT and (S)Variants

Supplementary Table 2. Spike protein variant mutations. Mutation information was obtained from literature sources. Additional mutations were made (R682G, R683S, R685S, K986P, and V987P) to the furin cleavage site to prevent degradation of proteins by host cell protease. Del denotes for deletion.

	WT	Alpha (B.1.1.7)	Beta (B.1.351)	Gamma (P.1)	Delta (B.1.617.2)	Omicron (B.1.1.529)
18	L		F	F		
20	T			N		
26	P			S		
67	A					V
69-70	HV	del				del
80	D		A			
95	T					I
138	D			Y		
142	G				D	D
143-145	VYY					del
144	Y	del				
154	E				K	
190	R			S		
211	N					del
212	L					I
215	D		G			
242-244	LAL		del			
246	R		I			
339	G					D
371	S					L
373	S					P
375	S					F
417	K		N	T		N
440	N					K
446	G					S
452	L				R	
477	S					N
478	T					K
484	E		K	K	Q	A
493	Q					K
496	G					S
498	Q					R
501	N	Y	Y	Y		Y
505	Y					H
547	T					K
570	A	D				
614	D		G		G	G
655	H			Y		Y
679	N					K
681	P	H			R	H
701	A		V			
716	T	I				
764	N					K
796	D					Y
856	N					K
954	Q					H
969	N					K
981	L					F
982	S	A				
1027	T			I		
1071	Q				H	
1101	H				D	
1118	D	H				

Supplementary Table 3. Results from two prior tests detecting anti-SARS-CoV-2 antibodies (COV2G Siemens 1st Gen and EUROIMMUN EIA) for corresponding individual samples tested in Fig. 3d. Interpretation, indicated as either positive (+) or negative (-), denotes the presence of anti-SARS-CoV-2 antibodies.

	1	2	3	4	5	6	7	8	9	10
**COV2G Siemens 1st Gen**	> 20.00	> 20.00	> 20.00	> 20.00	> 20.00	> 20.00	> 20.00	> 20.00	> 20.00	> 20.00
**Interpretation**	+	+	+	+	+	+	+	+	+	+
**EUROIMMUN EIA**	8.095	6.882	8.097	8.097	8.041	8.023	8.095	8.095	8.032	8.087
**Interpretation**	+	+	+	+	+	+	+	+	+	+

	11	12	13	14	15	16	17	18	19	20
**COV2G Siemens 1st Gen**	> 20.00	> 20.00	> 20.00	> 20.00	> 20.00	> 20.00	> 20.00	> 20.00	> 20.00	> 20.00
**Interpretation**	+	+	+	+	+	+	+	+	+	+
**EUROIMMUN EIA**	8.094	8.096	8.097	8.096	8.095	8.094	8.093	8.095	8.096	8.096
**Interpretation**	+	+	+	+	+	+	+	+	+	+

	21	22	23	24	25	26	27	28
**COV2G Siemens 1st Gen**	> 20.00	> 20.02	> 20.01	> 20.00	> 20.00	> 20.00	> 20.00	> 20.00
**Interpretation**	+	+	+	+	+	+	+	+
**EUROIMMUN EIA**	8.093	8.094	7.832	8.094	8.095	8.094	8.095	8.087
**Interpretation**	+	+	+	+	+	+	+	+

	29	30	31	32	33	34	35	36	37	38
**COV2G Siemens 1st Gen**	0	0	0.01	0.02	0	0	0	0.02	0	0.05
**Interpretation**	-	-	-	-	-	-	-	-	-	-
**EUROIMMUN EIA**	0.144	0.164	0.219	0.405	0.234	0.142	0.109	0.135	0.158	0.255
**Interpretation**	-	-	-	-	-	-	-	-	-	-

	39	40	41	42	43
**COV2G Siemens 1st Gen**	0	0.01	0	0	0
**Interpretation**	-	-	-	-	-
**EUROIMMUN EIA**	0.315	0.104	0.103	0.38	0.157
**Interpretation**	-	-	-	-	-

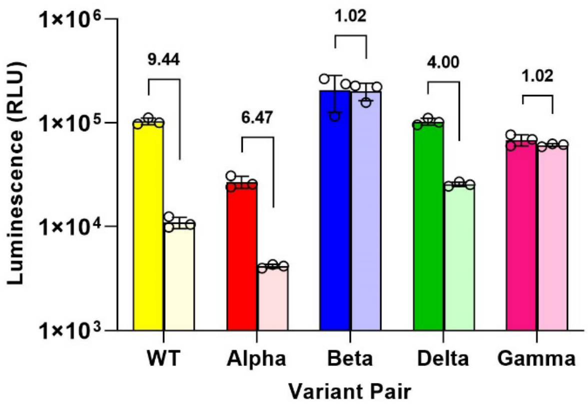

Supplementary Figure 2. Neutralization of full S protein variant pairs. The efficacy of 100 µg/mL NAb (Sino Biological, 40592-R001 - right of each pair) was tested with each variant pairs (darker color on left is value in absence of Nab). The Alpha variant showed the most decrease in signal followed by the Delta strain. Almost no neutralization was observed with the Beta or Gamma strain. Values above pairs show fold change. Source data are provided as a Source Data file.

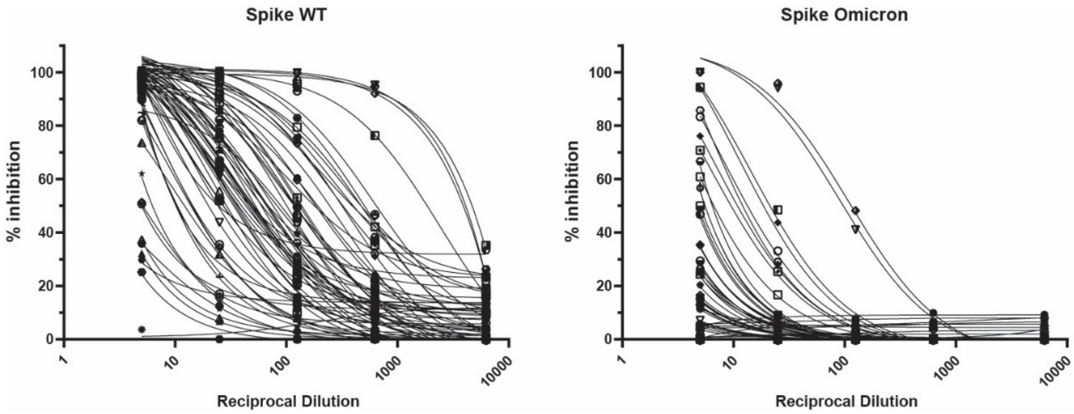

Supplementary Figure 3. 5-point curves of percentage of inhibition of all sample provided (n=66) against WT and Omicron trimeric spike protein. The data was fitted using a three-parameter logistic non-linear regression. Source data are provided as a Source Data file.

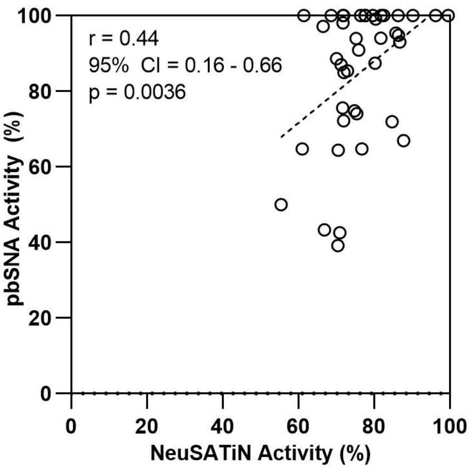

Supplementary Figure 4. Activity measured in protein-based surrogate neutralization assay (pbSNA) versus neu-SATiN for the Omicron variant shows moderate correlation, with a Pearson's r value of 0.44. The correlation is limited by the low number of samples containing neutralizing antibodies.
