# Supplementary material for: Homogeneous surrogate virus neutralization assay to rapidly assess neutralization activity of anti-SARS-CoV-2 antibodies
Source: Nat Commun. 2022 Jul 1;13:3716. doi: 10.1038/s41467-022-31300-9 (PMC9249905; doi:10.1038/s41467-022-31300-9)
Supplement: Supplementary file 3 — Reporting Summary [file 41467_2022_31300_MOESM3_ESM.pdf]

nature portfolio

Corresponding author(s): Shawn C Owen ; Igor Stagljar

Last updated by author(s): Apr 10, 2022

Reporting Summary

Nature Portfolio wishes to improve the reproducibility of the work that we publish. This form provides structure for consistency and transparency in reporting. For further information on Nature Portfolio policies, see our Editorial Policies and the Editorial Policy Checklist.

Statistics

For all statistical analyses, confirm that the following items are present in the figure legend, table legend, main text, or Methods section.

n/a	Confirmed	
☐	☒	The exact sample size (*n*) for each experimental group/condition, given as a discrete number and unit of measurement
☐	☒	A statement on whether measurements were taken from distinct samples or whether the same sample was measured repeatedly
☒	☐	The statistical test(s) used AND whether they are one- or two-sided<br>*Only common tests should be described solely by name; describe more complex techniques in the Methods section.*
☐	☒	A description of all covariates tested
☒	☐	A description of any assumptions or corrections, such as tests of normality and adjustment for multiple comparisons
☐	☒	A full description of the statistical parameters including central tendency (e.g. means) or other basic estimates (e.g. regression coefficient) AND variation (e.g. standard deviation) or associated estimates of uncertainty (e.g. confidence intervals)
☐	☒	For null hypothesis testing, the test statistic (e.g. *F*, *t*, *r*) with confidence intervals, effect sizes, degrees of freedom and *P* value noted<br>*Give P values as exact values whenever suitable.*
☒	☐	For Bayesian analysis, information on the choice of priors and Markov chain Monte Carlo settings
☒	☐	For hierarchical and complex designs, identification of the appropriate level for tests and full reporting of outcomes
☐	☒	Estimates of effect sizes (e.g. Cohen's *d*, Pearson's *r*), indicating how they were calculated

Our web collection on statistics for biologists contains articles on many of the points above.

Software and code

Policy information about availability of computer code

Data collection	TECAN Infinite M1000Pro
Data analysis	GraphPad Prism 9.0

For manuscripts utilizing custom algorithms or software that are central to the research but not yet described in published literature, software must be made available to editors and reviewers. We strongly encourage code deposition in a community repository (e.g. GitHub). See the Nature Portfolio guidelines for submitting code & software for further information.

Data

Policy information about availability of data

All manuscripts must include a data availability statement. This statement should provide the following information, where applicable:

- Accession codes, unique identifiers, or web links for publicly available datasets
- A description of any restrictions on data availability
- For clinical datasets or third party data, please ensure that the statement adheres to our policy

Source data are provided with this paper

Field-specific reporting

Life sciences study design

All studies must disclose on these points even when the disclosure is negative.

Sample size	Sample sizes are reported and determined by the availability of samples.
Data exclusions	All data are included.
Replication	All Experiments were repeated 2-4 times. All attempts at replication were successful
Randomization	Patient serum samples were selected randomly from respective cohorts (e.g. vaccinated, infected, etc). Covariants are not relative to this study.
Blinding	Investigators were blinded for experiments using patient serum samples, including both neu-SATiN and neutralizing antibody assays. Conditions for assay development were known when preparing samples and analyzing results.

Reporting for specific materials, systems and methods

We require information from authors about some types of materials, experimental systems and methods used in many studies. Here, indicate whether each material, system or method listed is relevant to your study. If you are not sure if a list item applies to your research, read the appropriate section before selecting a response.

Materials & experimental systems

n/a	Involved in the study	
☐	☒	Antibodies
☐	☒	Eukaryotic cell lines
☒	☐	Palaeontology and archaeology
☒	☐	Animals and other organisms
☐	☒	Human research participants
☒	☐	Clinical data
☒	☐	Dual use research of concern

Methods

n/a	Involved in the study	
☒	☐	ChIP-seq
☒	☐	Flow cytometry
☒	☐	MRI-based neuroimaging

Antibodies

Antibodies used	40592-MM57 (used for RBD pair screening) and 40592-R001 (used for WT pair screening) were both purchased from Sino Biological. Regn10933 (CPC511A), Regn10987 (CPC512A) and JS016 (CPC516A) were purchased from Cell Sciences
Validation	Antibodies were validated in previous reports. 40592-MM57: Asaka MN, Utsumi D, Kamada H, et al. Highly susceptible SARS-CoV-2 model in CAG promoter-driven hACE2-transgenic mice. JCI Insight. 2021;6(19):e152529. Published 2021 Oct 8. doi:10.1172/jci.insight.152529. 40592-R001: Guzmán-Martínez, O., Guardado, K., Varela-Cardoso, M. et al. Generation and persistence of S1 IgG and neutralizing antibodies in post-COVID-19 patients. Infection (2021). https://doi.org/10.1007/s15010-021-01705-7

Eukaryotic cell lines

Policy information about cell lines

Cell line source(s)	HEK 293 cell line was kindly provided by Prof. Jason Moffat at University of Toronto and was originally purchased from ATCC (CRL-1573). CaLu-3 cells were purchased from ATCC (HTB-55).
Authentication	Authentication was provided by the ATCC repository.
Mycoplasma contamination	The cell lines were not tested for mycoplasma.
Commonly misidentified lines (See ICLAC register)	No commonly misidentified cell lines were used.

Human research participants

Policy information about studies involving human research participants

Population characteristics	All samples were de-identified
Recruitment	No participants were enrolled - all samples were preexisting.
Ethics oversight	Samples were obtained from either the University of Utah School of Medicine, ARUP Laboratories, or from Unity Health. ARUP Laboratories (IRB approved protocol 0007740); Unity Health (REB approved protocol REB 20-107, Toronto)

Note that full information on the approval of the study protocol must also be provided in the manuscript.
